# PReS-FINAL-2033: Increase of adverse mild and moderate adverse events among patients with juvenile idiopathic arthritis treated with biological therapies

**DOI:** 10.1186/1546-0096-11-S2-P46

**Published:** 2013-12-05

**Authors:** CA Guillen Astete, L García Campayo, B Conde Campayo, A Sifuentes Giraldo, ML Gamir Gamir

**Affiliations:** 1 Pediatric Rheumatology Unit, Madrid, Spain; 2 Ramon y Cajal University Hospital, Madrid, Spain

## Introduction

The introduction of biological therapies (BT) has improve the management of patients with Juvenile Idiopathic Arthritis (JIA) in terms of relive of symptoms, enhancing the quality of live and reducing joint damage. Its pharmacological activity induces immunity transitory suppression that supposes an increased risk of acquire infectious diseases or a major severity of them. TB adverse events registries have painted special attention to severe events that conditioned in bed stay at hospitals or mortality and most of them are focused on adult cohorts. Paediatric BT adverse events registries are significantly fewer compared to adults and the recount of mild and moderate adverse events (according to OMERACT-8 definitions), which are fortunately highly frequent than others, have not properly compared with healthy children.

## Objectives

The purpose of this study is to determine the increase of risk of develop a mild or moderate adverse event in patients with JIA receiving BT compared to healthy people.

## Methods

We conducted a retrospective cohort study. Complete medical records of patients with JIA treated with BT since 2002 in our unit were revised. Among this group we included those patients who started BT before 16 years old. Control cohort was composed by healthy subjects same age and sex. Controls were obtained randomly from two different primary care units related to our hospital until complete three subjects for each case. The recount of mild and moderate adverse events was performed since starting BT in our cohort and at the same age in controls. All recounts finalized when BT was stopped of when subjects (patients or not) reached 16 years old or at December 31th, 2012.

## Results

Thirty patients were followed up during an average of 4.2 SD 1.8 years. Time of effective exposure to BT, excluding periods of stop of prescription was 3.1 SD 1.5 years. Healthy subjects were followed up an average of 4.8 SD 1.4 years. Incidence of mild adverse effects was 1.9 cases/patient/year in the BT group and 1.05 cases/subject/year into the control group (p < 0.05) which represents a risk increase of 80.9%. Incidence of moderate adverse events was 1.56 cases/patient/year in the BT group and 1.26 cases/patient/year into the control group (p = 0.04) which represents a risk increase of 23.8%.

## Conclusion

OMERACT definition of mild adverse events makes difficult its complete registration into medical records and it could underestimate its prevalence in healthy people compared with patients with JIA receiving BT who are under a close follow up. With this in mind we believe that the most valuable result is the risk increase of moderate adverse events due their need of medical approach (by definition). Considering that moderate adverse events are more frequent than severe ones, we consider this study provides relevant information to share with parents of patients who potentially will take BT. It is necessary a prospective follow up of major cohorts and over longer periods of time to obtain valuable information about risk increase among different groups of ages or different ILAR categories of JIA.

## Disclosure of interest

None declared.

